# Association of mannose-binding lectin, ficolin-2 and immunoglobulin concentrations with future exacerbations in patients with chronic obstructive pulmonary disease: secondary analysis of the randomized controlled REDUCE trial

**DOI:** 10.1186/s12931-021-01822-9

**Published:** 2021-08-14

**Authors:** Severin Vogt, Jörg D. Leuppi, Philipp Schuetz, Beat Mueller, Carmen Volken, Sarah Dräger, Marten Trendelenburg, Jonas Rutishauser, Michael Osthoff

**Affiliations:** 1 Division of Internal Medicine, University Hospital Basel, Petersgraben 4, 4031 Basel, Switzerland; 2 Department of Medicine, Kantonsspital Baselland, Liestal, Switzerland; 3 Medical University Department, Kantonsspital Aarau, Aarau, Switzerland; 4 Faculty of Medicine, University of Basel, Basel, Switzerland; 5 Central Laboratory, Kantonsspital Baselland, Bruderholz, Switzerland; 6 Department of Clinical Research, University of Basel, Basel, Switzerland; 7 Department of Medicine, Clinical Trial Unit, Kantonsspital Baden, Baden, Switzerland

**Keywords:** Mannose-binding lectin, Ficolin-2, Immunoglobulin, Chronic obstructive pulmonary disease, Exacerbation, Glucocorticoid treatment

## Abstract

**Background:**

The innate and adaptive immune system is involved in the airway inflammation associated with acute exacerbations in patients with chronic obstructive pulmonary disease (COPD). We evaluated the association of mannose-binding lectin (MBL), immunoglobulin (Ig) and ficolin-2 concentrations with COPD exacerbations and according to the glucocorticoid treatment duration for an index exacerbation.

**Methods:**

Post-hoc analysis of the randomized, double-blind, placebo-controlled REDUCE trial of 5 vs. 14 days of glucocorticoid treatment for an index exacerbation. MBL, ficolin-2 and total IgG/IgA and subclass concentrations were determined in stored samples drawn (n = 178) 30 days after the index exacerbation and associated with the risk of re-exacerbation during a 180-day follow-up period.

**Results:**

IgG and subclass concentrations were significantly lower after 14 days vs. 5 days of glucocorticoid treatment. Patients with higher MBL concentrations were more likely to suffer from a future exacerbation (multivariable hazard ratio 1.03 per 200 ng/ml increase (95% confidence interval (CI) 1.00–1.06), p = 0.048), whereas ficolin-2 and IgG deficiency were not associated. The risk was most pronounced in patients with high MBL concentrations, IgG deficiency and 14 days of glucocorticoid treatment pointing towards an interactive effect of MBL and IgG deficiency in the presence of prolonged glucocorticoid treatment duration [Relative excess risk due to interaction 2.13 (95% CI − 0.41–4.66, p = 0.10)]. IgG concentrations were significantly lower in patients with frequent re-exacerbations (IgG, 7.81 g/L vs. 9.53 g/L, p = 0.03).

**Conclusions:**

MBL modified the short-term exacerbation risk after a recent acute exacerbation of COPD, particularly in the setting of concurrent IgG deficiency and recent prolonged systemic glucocorticoid treatment. Ficolin-2 did not emerge as a predictor of a future exacerbation risk.

**Supplementary Information:**

The online version contains supplementary material available at 10.1186/s12931-021-01822-9.

## Background

Chronic obstructive pulmonary disease (COPD) is the third leading cause of death according to the World Health Organization (WHO) [1]. Acute exacerbations are the main reason for hospitalization, deterioration of lung function and increased morbidity and mortality.

Exacerbations are characterized by significant airway inflammation that is likely orchestrated by members of the innate and adaptive immunity. Mannose-binding lectin (MBL) is a soluble pattern recognition receptor (PRR) of the innate immune system. Upon binding to carbohydrate-enriched regions of microorganisms, the lectin pathway of the complement system is activated and phagocytosis enabled. Individuals with distinctive single nucleotide polymorphisms of the *MBL2* gene lack multimeric forms required for complement activation [2]. In patients with chronic pulmonary disorders such as bronchiectasis or cystic fibrosis, MBL deficiency has been associated with more frequent exacerbations and faster decline in forced expiratory volume in 1 s [3, 4]. In COPD, data regarding the association of MBL deficiency with the risk of exacerbation are conflicting [5–8].

Ficolin-2 is an additional soluble PRR of the lectin pathway. Low ficolin-2 concentrations may be observed in patients with bronchiectasis and may facilitate their development [9]. Data on ficolin-2 and COPD exacerbations are lacking.

As part of the adaptive immune system, immunoglobulins (Ig) play a critical role in the prevention of respiratory tract infections [10, 11]. IgG subclass deficiency can be observed in up to 20% of COPD patients [12] and may be associated with airway obstruction [13]. Reduction in IgG subclass concentrations might be an independent risk factor for COPD exacerbation and hospitalization [12, 14, 15]. The impact of IgG or IgA subclass deficiency in the setting of MBL deficiency or low ficolin-2 concentrations is unknown.

We aimed to clarify the association of MBL and immunoglobulin deficiencies and ficolin-2 concentration, respectively, with COPD exacerbation risk in participants of a previously published randomized, placebo-controlled and double-blind trial comparing 5 vs. 14 days of glucocorticoid treatment for acute COPD exacerbations.

## Methods

We performed a post-hoc analysis of a subgroup of patients included in the REDUCE study [16] a randomized, double-blind, placebo-controlled and multicenter trial (ISRCTN registry; Identifier: ISRCTN19646069; http://www.isrctn.org). Serum samples from 178/311 participants drawn at a follow-up visit 30 days after the index COPD exacerbation were available for the present study. This time point was chosen as it represents a steady-state condition without relevant systemic inflammation [17, 18]. Samples had been stored at − 80 °C and had not been thawed before.

MBL- and ficolin-2 concentrations were measured with a commercially available enzyme-linked immunosorbent assay detecting oligomeric functional proteins following the instructions of the corresponding manuals (MBL: Bioporto Diagnostics, Denmark; ficolin-2: Hycult Biotech, Uden, the Netherlands). IgG and subclasses were measured on an Image analyser using Sanquin reagents and a Sanquin calibrator. Sanquin standard was used and values were traceable to the WHO 67/97 standard. IgA and subclasses measurement was performed with a commercially available liquid latex reagent kit using the Beckman Image 800 (Beckman Coulter) machine and Sanquin reagents.

A serum concentration < 500 ng/mL is a widely accepted cut-off to define MBL deficiency [19]. Since it has been shown that serum concentrations of ficolin-2 can differ substantially depending on the type of tube used or on the sampling and handling procedure [20], we calculated the cut-off concentration based on the ficolin-2 concentrations of the present study cohort, defining 25% quantile serum concentrations as cut-off (3985 ng/mL) [21, 22]. IgG and IgG subclass deficiency was defined as IgG concentration below the lower limit of normal range (IgG < 7.5 g/L, IgG1 < 4.9 g/L, IgG2 < 1.5 g/L, IgG3 < 0.2 g/L, and IgG4 < 0.08 g/L). The cut-off value of IgA was < 0.82 g/L and of IgA subclasses IgA1 < 0.6 g/L and IgA2 < 0.06 g/L [23].

Baseline data were assessed during the acute index exacerbation.

In line with the original trial, the primary endpoint of our study was COPD re-exacerbation after the index exacerbation within the follow-up period of 180 days. We further evaluated ICU admission and frequency of re-exacerbations as secondary endpoints.

The following subgroup analyses were performed for the primary outcome: the combined effect of MBL and total IgG concentration and the effect of MBL concentration according to the originally assigned glucocorticoid treatment duration (5 vs. 14 days).

The statistical analysis was performed using R (version 4.0.2). Data from the two treatment arms of the REDUCE trial were pooled. We compared categorical variables with the chi squared test and used the Wilcoxon rank sum (Man Whitney) test for quantitative variables. For all conducted analyses, a p-value less than 0.05 was considered statistically significant.

We analysed time to re-exacerbation with Kaplan Meier models and cox proportional hazards models. P-values for Kaplan Meier analysis were derived from the log rank test.

For multivariable analysis, cox proportional hazards model was used. We defined the following variables a priori as control variables for adjustment: Length of hospital stay, age, gender, COPD stage, treatment group (5 days vs. 14 days of glucocorticoid treatment) and need of oxygen supply. Proportional hazards assumption was tested based on Schoenfeld residuals. To test for effect modifiers we calculated the absolute excess risk due to interaction (AERI) based on Kaplan Meier estimates and the relative excess risk due to interaction (RERI) based on the hazard ratios (HR) derived from a multivariate cox proportional hazards [24]. Based on these estimates, we calculated the attributable proportion (AP) of joint effect.

## Results

For the present analysis, serum samples of 178 patients comprising 57% of the original trial population were available, including 96 (54%) patients who were treated for 14 days and 82 (46%) patients who were treated for 5 days with 40 mg prednisone daily for the index exacerbation.

The present patient sample was a representative subgroup of the entire REDUCE cohort with no significant differences in baseline characteristics observed (Additional file 1). Mean age of the patients was 69 years, the majority was male (65%) and suffered from severe COPD GOLD grade 3 (Table 1). As glucocorticoid treatment may influence IgG concentrations, we investigated them according to the glucocorticoid treatment duration. Median total IgG concentrations were significantly lower (total IgG, 8.8 g/L vs. 9.8 g/L, p < 0.01) and IgG deficiency was more prevalent in the 14 days glucocorticoid group. Regarding IgG subclasses, the most relevant differences were present in IgG1, IgG3 and IgG4, whereas serum concentrations of MBL and ficolin-2 were similar (Table 2). The effects of smoking status during follow-up (day 30) and of glucocorticoid pretreatment before study entry on lectin and IgG concentrations are reported in the supplement (Additional files 2 and 3).

**Table 1:** Baseline characteristics of the present cohort at the time of the index exacerbation

	Present cohort n = 178
Age, years, mean (range)	69 (42-91)
Female gender, n (%)	62 (35)
Oxygen saturation in %, mean (SD)	88 (7)
Systolic blood pressure in mmHg, mean (SD)	142 (26)
Heart rate, per minute, mean (SD)	93 (19)
Temperature (°C), mean (SD)	37.5 (84)
14 days glucocorticoid treatment n (%)	96 (54)
5 days glucocorticoid treatment n (%)	82 (46)
COPD GOLD grade, n (%)	
I	24 (13)
II	60 (34)
III	94 (53)
IV	0
Dyspnea Score (Scale 1-5), n (%)	
1	5 (3)
2	17 (10)
3	22 (12)
4	50 (28)
5	76 (43)
NA	8 (4)
Home oxygen requirement, n (%)	
Yes	21 (12)
No	156 (87)
NA	1 (1)
Smoking status, n (%)	
Active	80 (45)
Stopped	98 (55)
NA	0 (0)
Glucocorticoid treatment before admission (iv + oral), n (%)	
Yes	34 (19)
No	144 (81)
NA	0 (0)
Antibiotic treatment before admission, n (%)	
Yes	33 (19)
No	144 (80)
NA	1 (1)
*Outcome*	
Re-exacerbation within 180 days, n (%)	
Overall	65 (36)
5 days glucocorticoid group	24 (29), n=82
14 days glucocorticoid group	41 (43), n=96
Death within 180 days, n (%)	
Overall	7 (4)
5 days glucocorticoid group	3 (4), n=82
14 days glucocorticoid group	4 (4), n=96
Death or exacerbation within 180 days, n (%)	
Overall	67 (38)
5 days glucocorticoid group	24 (29), n=82
14 days glucocorticoid group	43 (45), n=96

Clinical characteristics were assessed when patients were admitted to the hospital

*SD* standard deviation, *COPD* Chronic obstructive pulmonary disease, *d* day, *NA* not available

**Table 2:** Differences in MBL, ficolin-2 and immunoglobulin subclass concentrations and deficiencies according to the duration of glucocorticoid treatment for the index exacerbation (14 days vs. 5 days)

	14d steroids	5d steroids	p-value*
MBL, median (IQR), ng/mL	1262 (2528)	1409 (2324)	0.95
MBL < 500 ng/mL, n (%)	32 (33)	24 (29)	0.67
Ficolin-2, median (IQR), ng/mL	5422 (3692)	5302 (2544)	0.70
Ficolin-2 < 25% Quantile, n (%)	25 (26)	20 (24)	0.94
Total IgG, median (IQR), g/L	8.8 (3.8)	9.8 (3.1)	< 0.01
IgG deficiency, n (%)	36 (38)	13 (16)	< 0.01
IgG1, median (IQR), g/L	5.0 (2.6)	6.2 (2.3)	< 0.01
IgG1 deficiency, n (%)	47 (49)	18 (22)	< 0.01
IgG2, median (IQR), g/L	2.7 (1.4)	2.9 (1.8)	0.13
IgG2 deficiency, n (%)	9 (9)	7 (9)	1.00
IgG3, median (IQR), g/L	0.412 (0.29)	0.47 (0.40)	0.02
IgG3 deficiency, n (%)	13 (14)	7 (9)	0.41
IgG4, median (IQR), g/L	0.302 (0.46)	0.41 (0.65)	0.04
IgG4 deficiency, n (%)	13 (14)	10 (12)	0.97
IgA, median (IQR), g/L	2.3 (1.7)	2.56 (1.3)	0.34
IgA deficiency, n (%)	6 (6)	1 (1)	0.18
IgA1, median (IQR), g/L	2.0 (1.5)	2.1 (1.1)	0.34
IgA1 deficiency, n (%)	6 (6)	1 (1)	0.18
IgA2, median (IQR), g/L	0.49 (0.42)	0.55 (0.39)	0.27

*p-value < 0.05 derived from the Man Whitney U-Test or the Chi-square test, where appropriate

### Risk of COPD re-exacerbation

65 patients (37%) re-exacerbated during the follow-up period of 180 days and thus met the primary endpoint (Table 3). Deficiencies in total IgG, IgG1, IgG2 and IgG3 were more frequently present in patients who exacerbated during follow-up, whereas the opposite was true for MBL deficiency (25% in patients who exacerbated vs. 35% in patients with no exacerbation); these differences were not statistically significant. MBL concentrations were higher in patients with re-exacerbation (median 1854 ng/ml vs. 1141 ng/ml, p = 0.047) (Table 3). Ficolin-2 concentrations and the frequency of ficolin-2 concentration < 3985 ng/ml were not associated with the risk of exacerbation.

**Fig. 1:**
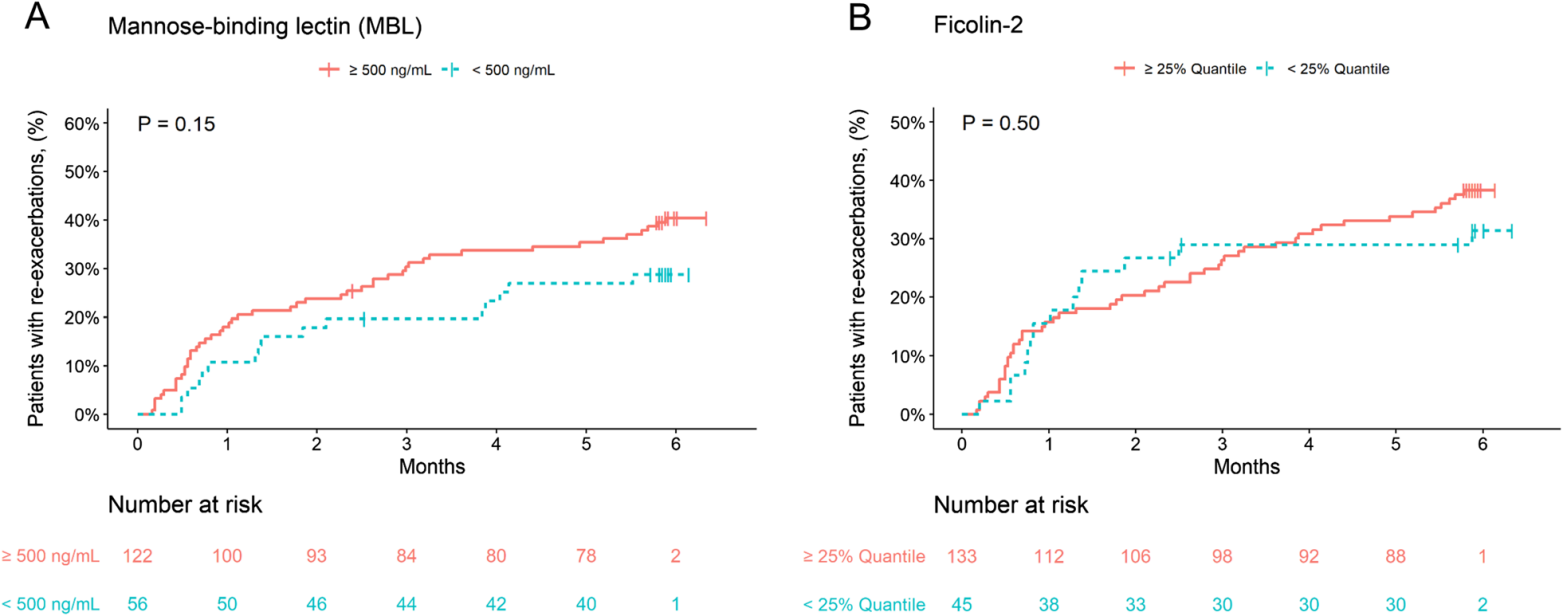
Time to re-exacerbation of COPD according to the presence of **A** MBL and **B** ficolin-2. P values were calculated with the log-rank test

**Fig. 2:**
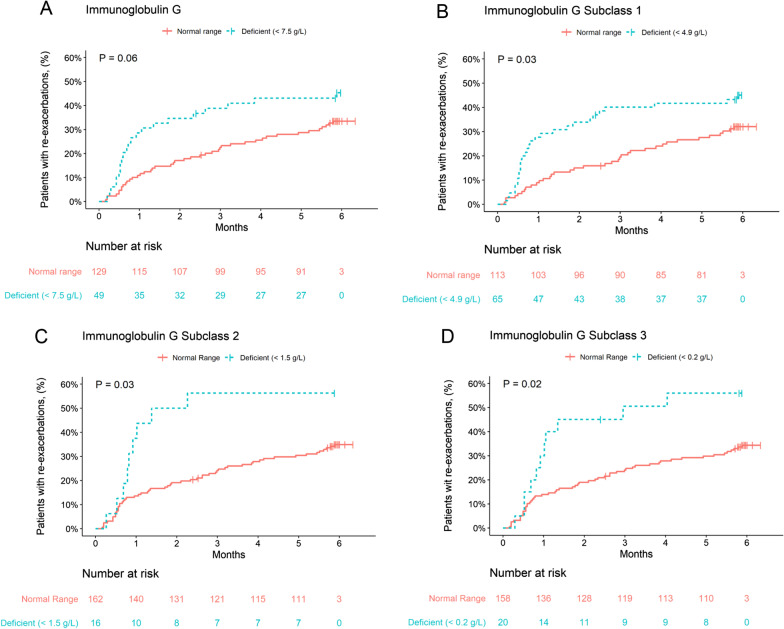
Time to re-exacerbation of COPD according to the presence of **A** immunoglobulin total and **B**-**D** subclass deficiencies. P values were calculated with the log-rank test

**Fig. 3:**
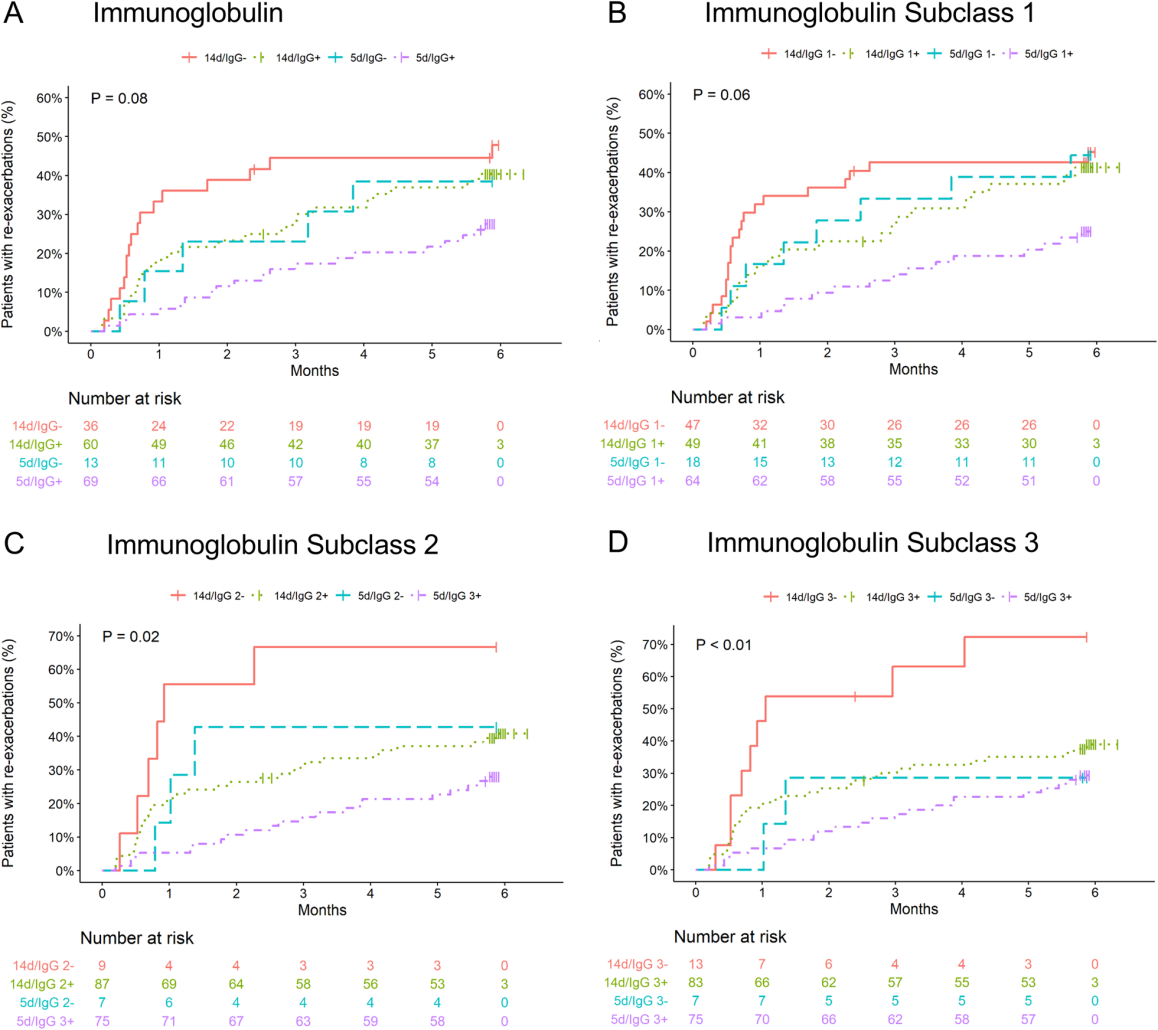
Time to re-exacerbation of COPD according to the presence of **A** immunoglobulin total and **B**–**D** subclass deficiencies and stratified according to the previous glucocorticoid treatment duration (5 days vs. 14 days). P-values were calculated with the log-rank test. IgG + and IgG1-3 + denote systemic concentrations of immunoglobulins equal to or above the respective cut-offs and IgG– and IgG1-3– below the cut-offs

Re-exacerbation during follow-up occurred numerically less frequently in MBL deficient patients (29% vs. 40%, p = 0.14) and more often in IgG deficient patients (45% vs. 33%, p = 0.06) (Figs. 1 and 2). Moreover, the primary endpoint occurred more frequently in patients with IgG1, -2, and -3 subclass deficiency, whereas no significant difference was observed in IgG4 deficient patients (Table 3). Low IgA and ficolin-2 concentrations were not associated with the risk of future exacerbations (data not shown). Analyses stratified for the treatment duration are shown in Fig. 3.

**Table 3:** Association of MBL, ficolin-2, and immunoglobulin concentrations and deficiencies with the primary and secondary endpoints

	Number of patients	Re-exacerbation within 180 days		Admission to ICU		> 1 exacerbation within 180 days	
		Yes	No	Yes	No	Yes	No
Total	N = 178	N = 65	N = 113	N = 24	N = 154	N = 22	N = 156
MBL, median (IQR), ng/mL	1286 (2427)	1854 (2390)	1141 (2462)*	2210 (2702)	1232 (2353)*	1539 (2231)	1344 (2471)
MBL < 500 ng/mL, n (%)	56 (31)	16 (25)	40 (35)	5 (21)	51 (33)	4 (18)	52 (33)
Ficolin-2, median (IQR), ng/mL	5338 (2949)	5705 (3330)	5245 (3004)	5275 (2523)	5325 (3030)	5226 (3086)	5339 (2890)
Ficolin-2 < 3985 ng/mL, n (%)	45 (25)	14 (22)	31 (27)	6 (25)	39 (25)	6 (27)	39 (25)
IgG total, median (IQR), g/L	9.2 (3.8)	8.8 (4.5)	9.4 (2.9)	8.7 (4.7)	9.3 (3.7)	7.8 (3.1)	9.5 (3.7)*
IgG deficiency, n (%)	49 (28)	22 (34)	27 (24)	9 (38)	40 (26)	10 (45)	39 (25)
IgG1, median (IQR), g/L	5.5 (2.7)	5.1 (3.4)	5.7 (2.2)	5.2 (3.0)	5.5 (2.7)	4.6 (1.3)	5.6 (2.7)*
IgG1 deficiency, n (%)	65 (37)	29 (45)	36 (32)	12 (50)	53 (34)	14 (64)	51 (33)*
IgG2, median (IQR), g/L	2.8 (1.7)	2.7 (1.9)	2.8 (1.6)	2.5 (1.5)	2.8 ( 1.7)	2.1 (1.8)	2.8 (1.6)
IgG2 deficiency, n (%)	16 (9)	9 (14)	7 (6)	1 (4)	15 (10)	4 (18)	12 (8)
IgG3, median (IQR), g/L	0.45 (0.35)	0.44 (0.4)	0.46 (0.32)	0.44 (0.35)	0.45 (0.35)	0.34 (0.24)	0.46 (0.35)*
IgG3 deficiency, n (%)	20 (11)	11 (17)	9 (8)	5 (21)	15 (10)	3 (14)	17 (11)
IgG4, median (IQR), g/L	0.34 (0.49)	0.27 (0.37)	0.39 (0.57)	0.41 (0.39)	0.33 (0.58)	0.18 (0.23)	0.38 (0.56)
IgG4 deficiency, n (%)	23 (13)	10 (15)	13 (12)	3 (12)	20 (13)	2 (9)	21 (13)
IgA total, median (IQR), g/L	2.5 (1.5)	2.6 (1.4)	2.4 (1.5)	2.4 (1.2)	2.5 (1.6)	2.3 (1.1)	2.5 (1.6)
IgA deficiency, n (%)	7 (4)	3 (5)	4 (4)	1 (4)	6 (4)	2 (9)	5 (3)
IgA1, median (IQR), g/L	2.03(1.23)	2.11 (1.37)	1.90 (1.24)	2.1 (1.1)	2.0 (1.3)	1.9 (0.9)	2.1 (1.4)
IgA1 deficiency, n (%)	7 (4)	3 (5)	4 (4)	1 (4)	6 (4)	2 (9)	5 (3)
IgA2, median (IQR), g/L	0.53 (0.44)	0.54 (0.43)	0.52 (0.42)	0.53 (0.45)	0.54 (0.41)	0.45 (0.22)	0.54 (0.45)

*Significant (< 0.05) p-values derived from Mann–Whitney U-test or Chi-square test, where appropriate

*MBL* mannose-binding lectin, *IQR* interquartile range, *ICU* intensive care unit, *Ig* immunoglobulin

After adjustment for potential confounders, the effect of MBL deficiency on the primary outcome became stronger [HR 0.56 (95% CI, 0.31, 1.01), p = 0.053] (Table 4). When used as a continuous variable, higher MBL concentrations were significantly associated with a higher risk of re-exacerbation in both the univariate and multivariate analysis [multivariate, HR 1.03 per 200 ng/mL increase in MBL concentration (95% CI, 1.00, 1.06), p = 0.048]. In contrast to the univariate analysis, IgG1, IgG2, and IgG3 deficiency were not identified as independent predictors of future exacerbations after adjustment. Further analyses taking into account the smoking status during follow-up and antibiotic pretreatment are reported in the supplement (Additional files 4 and 5).

### Secondary outcomes

**Fig. 4:**
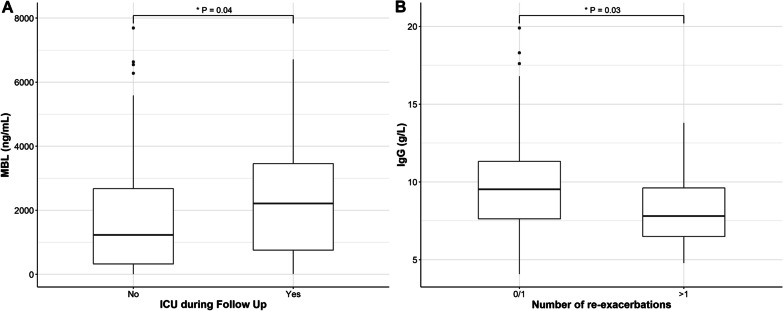
**A** Association of MBL concentrations with admission to an intensive-care unit during follow-up. **B** Association of IgG concentrations with the occurrence of frequent (> 1) exacerbations during follow-up

In total, 24 patients (13%) were admitted to ICU and more than one exacerbation occurred in 22 (12%) patients during follow-up. Patients admitted to the ICU had higher MBL concentrations than those who were not (median 2210 ng/mL vs. 1232 ng/mL, p = 0.04) (Fig. 4a), whereas other parameters measured were not associated with ICU admission. Only total IgG, IgG1 and IgG3 concentrations were significantly lower in patients who experienced more than one COPD re-exacerbation during follow-up (Table 3 and Fig. 4b). The frequency of MBL deficiency was 35% (40/113), 28% (12/43) and 18% (4/22), respectively, in patients experiencing no, one and more than one exacerbation during follow-up (p = 0.24).

### Joint effect and risk of re-exacerbation

**Fig. 5:**
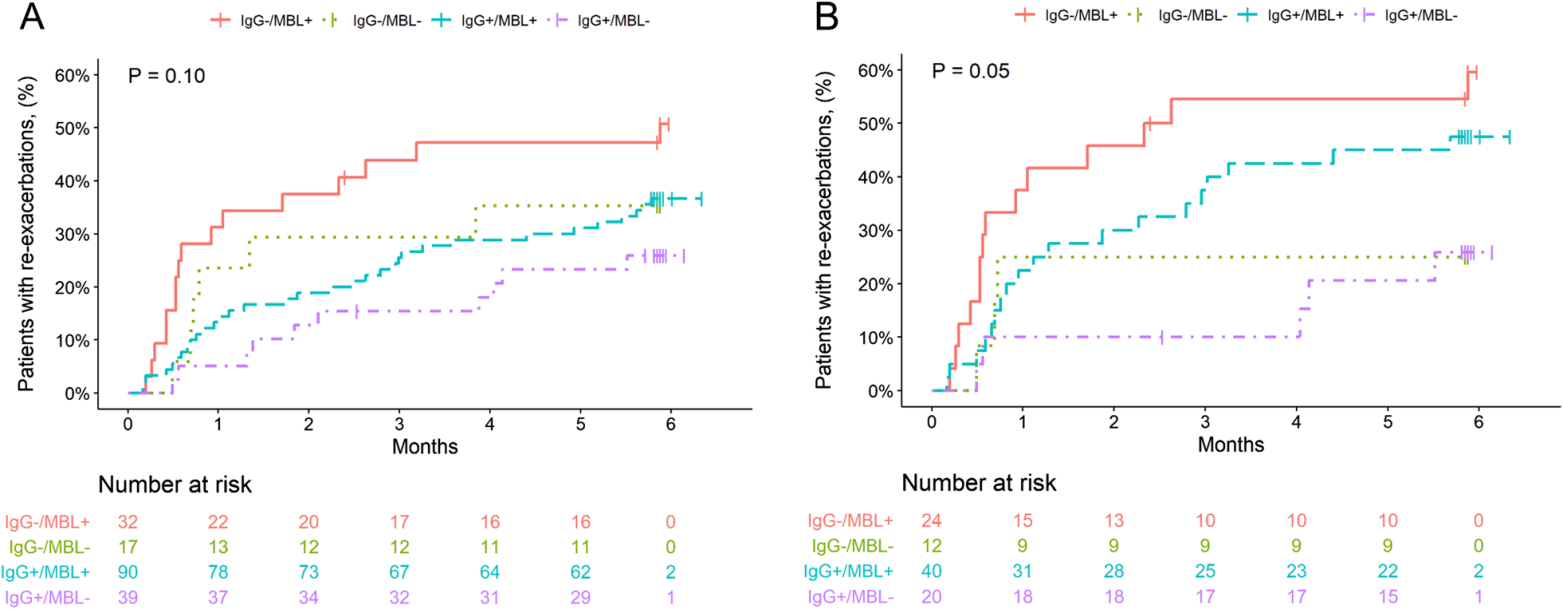
Time to re-exacerbation of COPD according to the concurrent presence of MBL deficiency and hypogammaglobulinemia. **A** entire cohort. **B** Patients treated with 14 days of glucocorticoid during index exacerbation. P-values were calculated with the log-rank test. MBL + denotes systemic concentrations of MBL ≥ 500 ng/mL and MBL – denotes systemic concentrations of MBL < 500 ng/ml. Likewise, IgG + and IgG1-3 + denote systemic concentrations of immunoglobulins equal to or above the respective cut-offs and IgG– and IgG1-3– below the cut-offs

Patients with MBL concentrations ≥ 500 ng/mL and low IgG concentrations (MBL+/IgG− ) re-exacerbated most frequently (51%), followed by patients with MBL deficiency and low IgG (MBL-/IgG−, 37%) and MBL sufficient patients with normal IgG (MBL + /IgG + , 35%). MBL deficiency combined with normal IgG (MBL-/IgG +) was associated with the lowest risk for re-exacerbation (26%) (Fig. 5a). The combined effect of MBL+/IgG− on re-exacerbation risk was mainly of additive nature. However, an absolute AERI of + 5% pointed towards an additional slight interactive effect. The estimated attributable proportion of joint effect due to interaction, which reflects the contribution of the interaction to the whole combined effect (e.g., additive and interactive), was 19%. After adjustment for the pre-specified control variables, we calculated the RERI based on the estimated hazard ratios for MBL, IgG and their interaction. A RERI of 0.81 (95% CI, − 1.14, 2.76, p = 0.41) for MBL+/IgG− pointed towards a minor interactive effect of MBL+/IgG− (RERI > 0), though not statistically significant.

### Influence of glucocorticoid treatment duration

In the 14-days glucocorticoid group, MBL deficiency and MBL concentrations independently predicted a lower re-exacerbation risk [multivariate cox regression, MBL < 500 ng/mL, HR 0.35 (95% CI 0.16, 0.79), p = 0.01; MBL (concentrations), HR 1.05 per 200 ng/mL increase (95% CI 1.02, 1.08), p ≤ 0.01]. MBL deficiency was less frequently observed amongst patients with frequent re-exacerbations [1 (7%) vs. 31 (38%) in patients without frequent re-exacerbations, p = 0.02].

The joint effect of MBL + and IgG− was considerably stronger in the 14 days glucocorticoid treatment arm. Especially MBL + patients frequently re-exacerbated (MBL+/IgG− ; 60%, MBL + /IgG + : 47%), whereas MBL deficient patients had a better outcome (MBL−/IgG+; 26%, MBL−/IgG−; 25%) (Fig. 5b). In addition, there was evidence for a moderate to strong interactive effect of MBL + and IgG− on re-exacerbation risk, reflected by an AERI of 13%. The attributable proportion due to interaction (AP) was 38%, suggesting a considerable contribution of the interaction to the joint effect of MBL + and IgG−. The strong interactive effect between MBL + and IgG− persisted after adjustment in a multivariate cox regression model. The RERI was 2.13 (95% CI − 0.41, 4.66 p = 0.10), pointing towards a much stronger interactive effect in this subgroup. The attributable proportion was 68%, indicating a major contribution of an interactive effect between MLB+/IgG− on the re-exacerbation risk.

None of these associations were observed in the short-treatment group pointing towards an important interactive effect of MBL with glucocorticoid treatment duration.

## Discussion

In the present study, the association of two PRR of the lectin pathway of the complement system and immunoglobulin deficiencies with future exacerbations was investigated in COPD patients who had recently been treated with prednisone for 5 or 14 days for an acute exacerbation. Our results demonstrate that low systemic MBL but not ficolin-2 concentrations are associated with a decreased risk of exacerbations during a 6-month follow-up period.

Previous studies have yielded conflicting results regarding the association of MBL and the risk of COPD exacerbations. While initial smaller studies had reported a positive association of genotypic MBL deficiency with the frequency of future exacerbations [5, 6, 25], a recent large study (n = 1796) has demonstrated the opposite effect, i.e. a significant lower frequency of exacerbations in patients with genotypic MBL deficiency [26]. Results from our study complement these results by showing for the first time that phenotypic MBL deficiency (< 500 ng/mL) is associated with a reduced risk for exacerbation after adjustment for covariates. Previous studies assessing MBL concentrations have only reported the association with frequent vs. infrequent exacerbations without employing a time-dependent model [6, 7]. Whereas Mandal et al. reported no difference in MBL concentrations [6], the prevalence of phenotypic MBL deficiency was numerically lower in patients with frequent exacerbations in the study by Eagan et al. [7], which supports our findings.

The association of MBL deficiency with a reduced risk for exacerbations is surprising at first glance given the reported association of MBL deficiency with the risk for infections [19, 27]. Moreover, airway inflammation may theoretically be increased given the fact that MBL has been shown to promote non-inflammatory sequestration of dying cells [28]. However, as a double-edged sword MBL has also been previously recognized to significantly augment the inflammatory response eliciting collateral damage [29]. Indeed, oxidative stress in the airways of COPD patients has been shown to modify the effects of MBL, leading not only to a loss of function but, even more important, to an inhibitory effect on the phagocytosis of microorganisms and on the efferocytosis of apoptotic bronchial epithelial cells by human alveolar macrophages [30]. Consequently, this inhibitory effect may promote an excessive inflammatory response to apoptotic cells or pathogens. Low MBL concentrations may be advantageous in this setting as the amount of oxidized MBL proteins capable of inhibiting macrophages is probably lower in the airways of these patients.

Our study is unique in that the association of MBL deficiency with the risk of COPD exacerbation was also assessed according to the duration of previous glucocorticoid treatment and according to the concomitant presence of hypogammaglobulinemia. Interestingly, the association of MBL deficiency with a reduced exacerbation risk was most pronounced in the absence of hypogammaglobulinemia in patients who had received a 14-day glucocorticoid treatment (compared to only 5 days). Conversely, patients with normal or high MBL concentrations but with hypogammaglobulinemia had the highest risk for additional exacerbations. High MBL concentrations particularly increased re-exacerbation risk in the 14-day glucocorticoid group implying a relevant interactive effect (i.e., more than additive) of MBL in the presence of additional risk factors. In accordance, MBL deficiency is a disease modifier primarily when adaptive immunity is either immature (e.g. during early childhood [31] or compromised [27]). As such, systemic glucocorticoid treatment may impair adaptive immunity, and both hypogammaglobulinemia and systemic glucocorticoid treatment may increase susceptibility to future microbial colonization and infection of the respiratory tract [32, 33] by reducing IgG related complement activation, phagocytosis and antibody-dependent cell-mediated cytotoxicity [34]. In addition, systemic glucocorticoid treatment promotes neutrophilia, which may lead to excessive inflammation in the presence of MBL due to an inadequate removal of dead neutrophils [35]. In line, the observed highest risk for exacerbations in patients with high MBL and low IgG concentrations may reflect an incremental impairment of phagocytosis of pathogens (in particular in patients colonized/infected with *Haemophilus influenzae *[8]) or dying cells by hypogammaglobulinemia and oxidized MBL. Interestingly, a recent large study identified hypogammaglobulinemia as risk factor for future COPD related hospitalizations only in patients with a previous history of COPD hospitalization [15], which exactly mirrors the present patient cohort. Hence, the results from our study support short-term glucocorticoid for acute exacerbations.

Despite the association of ficolin-2 with the development of bronchiectasis [9] and its participation in the silent removal of dying host cells [36], we found no association with the risk of future exacerbations in COPD.

Hypogammaglobulinemia and IgG1, -2, and -3 deficiencies, but not IgG4 and IgA deficiencies, were associated with an increased risk of exacerbations, however only in univariate analysis. Previous studies have demonstrated an increased risk for exacerbations and even hospitalization in COPD patients with hypogammaglobulinemia [15, 37], although the latter association was driven by patients with a history of previous COPD related admission. IgG1 and IgG2 deficiencies were associated with an increased risk of exacerbations in a COPD population from two randomized-controlled trials [12], as were low IgA concentrations in an observational COPD cohort [38]. Several reasons may account for the weaker association observed in our study. First, Ig concentrations were measured 30 days after the index exacerbation, and hence 2 to 3 weeks after the end of systemic glucocorticoid treatment, whereas they were measured at baseline in previous studies. Ig concentrations are significantly reduced during and even 3 weeks after the end of glucocorticoid treatment [39]. In line, IgG concentrations were significantly lower in the patient group having been treated for 14 days with systemic glucocorticoid recently. Second, different assays for the measurement of IgG subclass concentrations were used with different cut-offs suggested by the manufacturer (e.g. IgG1 < 4.9 g/L vs. < 2.8 g/L[12]) and the definition of hypogammaglobulinemia varied (from < 6.1 g/L to < 7.5 g/L [15, 40]). Third, adjustment may have been limited in the present study due to the rather small sample size. At present, it remains unclear if the significant degree of hypogammaglobulinemia observed in COPD patients is cause or effect of exacerbations (and their treatment with systemic glucocorticoids). If Ig deficiency is indeed a relevant risk factor for COPD exacerbations, replacement treatment with intravenous immunoglobulin (IVIG) preparations may be warranted. However, results of randomized trials are required before IVIG therapy can be incorporated into routine clinical practice. In contrast to MBL, which may be determined once during a stable disease period, the optimal time point of Ig measurement in order to predict future exacerbation risk is unknown.

Limitations of our study include the availability of study samples only from a subgroup of participants of the original randomized-controlled REDUCE trial. Follow-up was short, and analysis of the incidence rate ratios of exacerbations and long-term risk of MBL or Ig deficiencies was not possible. For ficolin-2, the lack of analysis of genetic data may preclude a definitive conclusion regarding its role in COPD.

## Conclusions

MBL modified the short-term exacerbation risk in our cohort of patients with a recent acute exacerbation of COPD, particularly in the setting of concurrent hypogammaglobulinemia and recent prolonged systemic glucocorticoid treatment. Ficolin-2 did not emerge as a predictor of a future exacerbation risk. If confirmed in intervention studies, the MBL concentration may be taken into account in COPD trials evaluating the benefit of immunoglobulin substitution treatment to prevent frequent exacerbations.

**Table 4:** Risk of exacerbation within 180 days as determined by uni- and multivariate cox regression analysis (adjustment for age, gender, oxygen supply, length of hospital stay, COPD grade and treatment group)

	Univariate cox regression		Multivariate cox regression	
	HR (CI 5%, CI 95%)	p-value	HR (CI 5%, CI 95%)	p-value
MBL (scaled per 200 ng/mL)	1.03 (1.00, 1.06)	0.02	1.03 (1.00, 1.06)	0.048
MBL < 500 ng/mL	0.65 (0.37, 1.15)	0.14	0.56 (0.31, 1.01)	0.053
Ficolin-2 (scaled per 200 ng/mL)	1.00 (0.99, 1.02)	0.65	1.01 (0.99, 1.03)	0.42
Ficolin-2 < 25% Quantile	0.82 (0.45, 1.48)	0.50	0.78 (0.43, 1.42)	0.42
IgG deficiency	1.62 (0.97, 2.71)	0.07	1.42 (0.83, 2.44)	0.21
IgA deficiency	1.36 (0.43, 4.34)	0.60	1.27 (0.39, 4.19)	0.69
Ig subclasses				
IgG 1 deficiency	1.69 (1.04, 2.76)	0.04	1.39 (0.82, 2.33)	0.22
IgG 2 deficiency	2.14 (1.05, 4.33)	0.04	1.98 (0.91, 4.31)	0.08
IgG 3 deficiency	2.09 (1.09, 4.00)	0.03	1.52 (0.76, 3.05)	0.23
IgG 4 deficiency	1.24 (0.63, 2.44)	0.53	1.5 (0.69, 3.27)	0.31
IgA 1 deficiency	1.36 (0.43, 4.34)	0.6	1.27 (0.39, 4.19)	0.69

*HR* hazard ratio, *CI* Confidence Interval, *MBL* mannose-binding lectin, *Ig* Immunoglobulin

## Supplementary Information

**Additional file 1.** Comparison of baseline characteristics of the present cohort with the entire REDUCE study cohort at the time of the index exacerbation according to the duration of glucocorticoid treatment for the index exacerbation (14 days vs. 5 days). Clinical characteristics were assessed when patients were admitted to the hospital.

**Additional file 2.** Influence of current smoking on day 30 on plasma lectin and immunoglobulin levels (as measured on day 30).

**Additional file 3.** Influence of glucocorticoid pretreatment on plasma lectin and immunoglobulin levels (as measured on day 30).

**Additional file 4.** Risk of exacerbation within 180 days as determined by multivariate Cox regression analysis taking into account current smoking status (adjustment for age, gender, oxygen supply, length of hospital stay, COPD grade treatment group and current smoking).

**Additional file 5.** Risk of exacerbation within 180 days as determined by multivariate Cox regression analysis taking into account antibiotic pretreatment (adjustment for age, gender, oxygen supply, length of hospital stay, COPD grade treatment group and antibiotic pretreatment).

## Data Availability

All data generated or analysed during this study are included in this published article (and its additional information files).

## Abbreviations

AERI: Absolute excess risk due to interaction
AP: Attributable proportion
BAL: Bronchoalveolar lavage
CI: Confidence Interval
COPD: Chronic obstructive pulmonary disease
FEV1: Forced Expiratory Volume in 1 s
HR: Hazard ratio
ICU: Intensive care unit
Ig: Immunoglobulin
IgA: Immunoglobulin A
IgG: Immunoglobulin G
IQR: Interquartile range
IVIG: Intravenous immunoglobulin
MBL: Mannose-binding lectin
PRR: Pattern recognition receptor
RERI: Relative excess risk due to interaction
SD: Standard deviation
WHO: World Health Organization
