# Supplementary material for: Association of mannose-binding lectin, ficolin-2 and immunoglobulin concentrations with future exacerbations in patients with chronic obstructive pulmonary disease: secondary analysis of the randomized controlled REDUCE trial
Source: Respir Res. 2021 Aug 14;22:227. doi: 10.1186/s12931-021-01822-9 (PMC8364051; doi:10.1186/s12931-021-01822-9)
Supplement: Supplementary file 1 — Additional file 1. Comparison of baseline characteristics of the present cohort with the entire REDUCE study cohort at the time of the index exacerbation according to the duration of glucocorticoid treatment for the index exacerbation (14 days vs. 5 days). Clinical characteristics were assessed when patients were admitted to the hospital. [file 12931_2021_1822_MOESM1_ESM.docx]

**Additional File 1**

Comparison of baseline characteristics of the present cohort with the entire REDUCE study cohort at the time of the index exacerbation according to the duration of glucocorticoid treatment for the index exacerbation (14 days vs. 5 days). Clinical characteristics were assessed when patients were admitted to the hospital.

| Baseline Characteristic | **Present cohort**  **n = 178** | | **Entire REDUCE study cohort**  **n = 311** | |
| --- | --- | --- | --- | --- |
|  | **Short term group (5d)**  **n = 82** | **Conventional group (14d)**  **n = 96** | **Short term group (5d)**  **n = 156** | **Conventional group (14d)**  **n = 155** |
| Age, years, mean (range) | 69 (42 – 91) | 69 (43 – 87) | 69 (42-97) | 69 (43 – 91) |
| Female sex, n (%) | 24 (29) | 38 (40) | 51 (33) | 72 (46) |
| Oxygen saturation in %, mean (SD) | 89 (7) | 87 (7) | 89 (8) | 88 (7) |
| Systolic blood pressure in mmHg, mean (SD) | 145 (26) | 139 (25) | 142 (25) | 141 (26) |
| Heart rate, per minute, mean (SD) | 93(18) | 92 (20) | 94 (19) | 93 (19) |
| Temperature (°C), mean (SD) | 37.5 (0.8) | 37.5 (0.9) | 37.4 (0.9) | 37.5 (0.9) |
|  |  |  |  |  |
| COPD GOLD grade, n (%) |  |  |  |  |
| 1I | 14 (17) | 10 (10) | 24 (15) | 17 (11) |
| II | 29 (35) | 31 (32) | 47 (30) | 49 (32) |
| III | 39 (48) | 55 (57) | 84 (54) | 85 (55) |
| IV | 0 (0) | 0 (0) | 1 (1) | 4 (3) |
| Dyspnea Score (Scale 1-5), n (%) |  |  |  |  |
| 1 | 2 (2) | 3 (3) | 4 (3) | 4 (3) |
| 2 | 9 (11) | 8 (8) | 13 (8) | 14 (9) |
| 3 | 13 (16) | 9 (9) | 23 (15) | 15 (10) |
| 4 | 24 (29) | 26 (27) | 46 (29) | 43 (28) |
| 5 | 32 (39) | 44 (46) | 63 (40) | 67 (43) |
| NA | 2 (2) | 6 (6) | 7 (4) | 12 (8) |
| Home oxygen requirement, n (%) |  |  |  |  |
| Yes | 10 (12) | 11 (11) | 24 (15) | 16 (10) |
| No | 72 (88) | 84 (88) | 131 (84) | 135 (87) |
| NA | 0 (0) | 0 (0) | 1 (1) | 4 (3) |
| Smoking status, n (%) |  |  |  |  |
| Active | 41 (50) | 39 (41) | 77 (49) | 62 (40) |
| Stopped | 41 (50) | 57 (59) | 79 (51) | 90 (58) |
| NA | 0 (0) | 0 (0) | 0 (0) | 3 (2) |
| Glucocorticoid treatment before admission (iv + oral), n (%) |  |  |  |  |
| Yes | 19 (23) | 15 (16) | 40 (26) | 31 (20) |
| No | 63 (77) | 81 (84) | 116 (74) | 121 (78) |
| NA | 0 (0) | 0 (0) | 0 (0) | 3 (2) |
| Antibiotic treatment before admission, n (%) |  |  |  |  |
| Yes | 17 (21) | 16 (17) | 32 (21) | 21 (14) |
| No | 64 (78) | 80 (83) | 121 (78) | 129 (83) |
| NA | 1 (1) | 0 (0) | 3 (2) | 5 (3) |
