# Supplementary material for: Association of mannose-binding lectin, ficolin-2 and immunoglobulin concentrations with future exacerbations in patients with chronic obstructive pulmonary disease: secondary analysis of the randomized controlled REDUCE trial
Source: Respir Res. 2021 Aug 14;22:227. doi: 10.1186/s12931-021-01822-9 (PMC8364051; doi:10.1186/s12931-021-01822-9)
Supplement: Supplementary file 2 — Additional file 2. Influence of current smoking on day 30 on plasma lectin and immunoglobulin levels (as measured on day 30). [file 12931_2021_1822_MOESM2_ESM.docx]

**Additional File 2**

Influence of current smoking on day 30 on plasma lectin and immunoglobulin levels (as measured on day 30).

|  | No smoking | Current smoking | p-value* |
| --- | --- | --- | --- |
| MBL, median (IQR), ng/mL | 1240 (2385) | 1500 (2235) | 0.32 |
| Ficolin-2, median (IQR), ng/mL | 5297 (3131) | 5478 (2249) | 0.40 |
| Total IgG, median (IQR), g/L | 9.53 (3.80) | 8.73 (3.36) | 0.54 |
|  |  |  |  |
| IgG1, median (IQR), g/L | 5.2 (2.80) | 5.45 (1.90) | 0.53 |
| IgG2, median (IQR), g/L | 2.78 (1.74) | 2.82 (1.39) | 0.92 |
| IgG3, median (IQR), g/L | 0.45 (0.29) | 0.45 (0.43) | 0.46 |
| IgG4, median (IQR), g/L | 0.34 (0.46) | 0.36 (0.61) | 0.66 |

*p-value derived from the Man Whitney U-Test

Abbreviations: Ig, immunoglobulin; IQR; interquartile range; MBL, mannose-binding lectin;
