# Supplementary material for: Association of mannose-binding lectin, ficolin-2 and immunoglobulin concentrations with future exacerbations in patients with chronic obstructive pulmonary disease: secondary analysis of the randomized controlled REDUCE trial
Source: Respir Res. 2021 Aug 14;22:227. doi: 10.1186/s12931-021-01822-9 (PMC8364051; doi:10.1186/s12931-021-01822-9)
Supplement: Supplementary file 3 — Additional file 3. Influence of glucocorticoid pretreatment on plasma lectin and immunoglobulin levels (as measured on day 30). [file 12931_2021_1822_MOESM3_ESM.docx]

**Additional File 3**

Influence of glucocorticoid pretreatment on plasma lectin and immunoglobulin levels (as measured on day 30).

|  | No pretreatment  with steroids | Pretreatment  with steroids | p-value* |
| --- | --- | --- | --- |
| MBL, median (IQR), ng/mL | 1264 (2417) | 1431 (2842) | 0.16 |
| Ficolin-2, median (IQR), ng/mL | 5293 (3026) | 6097 (2747) | 0.42 |
| Total IgG, median (IQR), g/L | 9.53 (3.40) | 7.78 (3.47) | 0.07 |
|  |  |  |  |
| IgG1, median (IQR), g/L | 5.64 (2.65) | 5.10 (2.31) | 0.25 |
| IgG2, median (IQR), g/L | 2.78 (1.52) | 2.96 (2.08) | 0.96 |
| IgG3, median (IQR), g/L | 0.46 (0.35) | 0.40 (0.28) | 0.25 |
| IgG4, median (IQR), g/L | 0.36 (0.51) | 0.27 (0.41) | 0.18 |

*p-value derived from the Man Whitney U-Test

Abbreviations: Ig, immunoglobulin; IQR; interquartile range; MBL, mannose-binding lectin;
