# Supplementary material for: Association of mannose-binding lectin, ficolin-2 and immunoglobulin concentrations with future exacerbations in patients with chronic obstructive pulmonary disease: secondary analysis of the randomized controlled REDUCE trial
Source: Respir Res. 2021 Aug 14;22:227. doi: 10.1186/s12931-021-01822-9 (PMC8364051; doi:10.1186/s12931-021-01822-9)
Supplement: Supplementary file 4 — Additional file 4. Risk of exacerbation within 180 days as determined by multivariate Cox regression analysis taking into account current smoking status (adjustment for age, gender, oxygen supply, length of hospital stay, COPD grade treatment group and current smoking). [file 12931_2021_1822_MOESM4_ESM.docx]

**Additional File 4**

Risk of exacerbation within 180 days as determined by multivariate Cox regression analysis taking into account current smoking status (adjustment for age, gender, oxygen supply, length of hospital stay, COPD grade treatment group and **current smoking**).

|  | Multivariate Cox Regression | |
| --- | --- | --- |
|  | HR  (CI 5%, CI 95%) | p-value |
| MBL (scaled per 200 ng/mL) | 1.03 (1.00, 1.05) | 0.06 |
| MBL<500 ng/mL | 0.56 ( 0.32, 0.99) | **0.047** |
| Ficolin-2 (scaled per 200 ng/mL) | 1.01 (0.99, 1.02) | 0.61 |
| Ficolin-2 < 25% Quantile | 0.84 (0.47, 1.49) | 0.55 |
| IgG deficiency | 1.46 (0.86, 2.48) | 0.16 |
| IgA deficiency | 1.38 (0.42, 4.57) | 0.60 |
|  |  |  |
| Ig subclasses |  |  |
| IgG 1 deficiency | 1.38 (0.83, 2.30) | 0.21 |
| IgG 2 deficiency | 1.84 (0.85, 4.00) | 0.13 |
| IgG 3 deficiency | 1.58 (0.81, 3.08) | 0.18 |
| IgG 4 deficiency | 1.50 (0.70, 3.23) | 0.31 |
| IgA 1 deficiency | 1.38 (0.42, 4.58) | 0.60 |

Abbreviations: CI; confidence interval; HR, hazard ratio; Ig, immunoglobulin; MBL, mannose-binding lectin;
